# Recurrent endobronchial occlusion and aorto-bronchial fistula formation in Behcet’s disease

**DOI:** 10.1186/s13019-023-02145-0

**Published:** 2023-01-13

**Authors:** Niloy Rahman, Eshan Senanayake, Jorge Mascaro, Deva Situnayake, Ehab S. Bishay, Akshay J. Patel

**Affiliations:** 1 Department of Thoracic Surgery, University Hospitals Birmingham NHS Trust, Birmingham, UK; 2 Department of Cardiac Surgery, University Hospitals Birmingham, Birmingham, UK; 3 Department of Rheumatology, National Behcet’s Centre for Excellence, Birmingham City Hospital, Birmingham, UK; 4 Institute of Immunology and Immunotherapy, University of Birmingham, Vincent Drive, Edgbaston, B15 2TT UK

**Keywords:** Behcet’s disease, Mouth and Genital ulcers with inflamed cartilage (MAGIC) syndrome, Endobronchial occlusion, Descending thoracic aortic aneurysm

## Abstract

**Background:**

Behcet’s disease is a multi-system inflammatory disorder. A small subset of patients with Behcet’s develop relapsing polychondritis which is classified as a separate disease known as Mouth and Genital ulcers with inflamed cartilage (MAGIC syndrome). It has previously been observed that this condition can also affect the cartilaginous tissue in the tracheobronchial tree.

**Case presentation:**

We present the case of a 44-year-old lady with Behcet’s Disease, Mouth and Genital ulcers with inflamed cartilage (MAGIC) syndrome and an aortic Frozen Elephant Trunk (FET) who presented to hospital with recurrent episodes of left lobar collapse of the lung. During bronchoscopy, we found the presence of multiple inflammatory endobronchial webs occluding segments of the left bronchial tree. Repeated examinations showed evidence that these inflammatory webs were progressing in size, density and location. Furthermore, we noticed herniation of her descending aortic FET into her left bronchial tree forming an aorto-bronchial fistula which was complicated by a graft infection. Her descending aortic FET section was surgically replaced with an open procedure and bronchoscopic interventions attempted to remove the occlusions in her bronchial tree. Despite optimisation of medical management and surgical correction, this patient continued to develop progressive occlusion of her left bronchial tree, resulting in a chronically collapsed left lung.

**Conclusions:**

A multi-disciplinary team approach is of paramount importance in order to optimally manage patients with Behcet’s disease, balancing immunosuppressive regimens that need close monitoring and titration in the context of potential surgical intervention and the risk for intercurrent infection.

## Introduction

Behcet’s disease is a multi-system inflammatory disorder originally described by Turkish dermatologist Hulusi Behcet in 1937. He reported on a series of three patients presenting with recurrent oral, genital and ocular lesions with no identifiable aetiology [1]. At the time it was proposed that the cause could be from a dental infection, even though no bacterial, fungal or viral agent could be found. Though the cause of Behcet’s remains unclear, we do know that the histological hallmarks of Behcet’s are that of a non-specific variable vessel vasculitis of small and large arteries and veins [2]. This includes both the systemic and pulmonary circulations. Intrathoracic vessels may show perivascular infiltration of lymphocytes and monocytes with severe inflammation of the tunica media which can predispose to the formation of aneurysms in Behcet’s disease.

Of particular interest to us are the thoracic complications of Behcet’s which are well documented in the literature. The prevalence of thoracic aortic aneurysms has been reported to be approximately 5% in cross sectional studies of Behcet’s patients [3]. Other documented complications include the development of pulmonary aneurysms, pulmonary infarction from recurrent in situ thromboses, pleural effusions, and thromboses of the great veins and aneurysms of the coronary arteries [4]. Rarely, a small subset of patients with Behcet’s also develop relapsing polychondritis [5] which is classified as a separate disease known as Mouth and Genital ulcers with inflamed cartilage (MAGIC syndrome). It has previously been observed that this condition can also affect the cartilaginous tissue in the tracheobronchial tree [6].

Treatment is tailored depending on the severity of the disease, according to the EULAR guidelines [7] but commonly involves use of multiple immunosuppressant agents including prednisolone, cyclophosphamide and biologics such as anti TNF targeted therapies and tocilizumab targeting the IL 6 pathway. Large aneurysms in Behcet’s patients can be managed surgically. Unfortunately, there is a significant rate of failure in this form of treatment due to the development of graft occlusion, stenosis, recurrent anastomotic aneurysms, infection, and A-V fistulas [4, 8]^**.**^ Suppression of disease activity prior to surgery is considered optimal in order to reduce the risks of these post-surgical complications.

In this report we will discuss the case of a 44 year old lady with Behcet’s who previously had a total replacement of her aortic arch with a FET due to severe aneurysmal disease. She was found to have an aorto-bronchial fistula due to her FET herniating into her left main bronchus. She was also found to have multiple endobronchial inflammatory webs causing recurrent lobar collapse.

## Case presentation

A 44-year-old lady with a 27-year history of Behcet’s disease presented to hospital with breathlessness secondary to recurrent collapse of her left lower lobe. Consequent to her underlying disease process, she had incurred numerous vascular complications, including a popliteal aneurysm, a femoral pseudo-aneurysms at previous arterial puncture site, and coronary and descending thoracic aortic aneurysms. She also had a history in keeping with relapsing polychondritis (MAGIC syndrome) and scleritis. Most significantly she received a total aortic arch replacement with a Frozen Elephant Trunk (FET) procedure in 2014 for treatment of extensive aortic aneurysmal disease. Prior to aortic arch replacement she had received treatment sequentially with high dose corticosteroid therapy, azathioprine or methotrexate therapy in combination with infliximab (anti-TNF therapy), pulsed intravenous cyclophosphamide therapy and subsequently B cell depletion therapy in the form of IV Rituximab. At this presentation her Behcet’s was managed with mycophenolate mofetil, IV monthly tocilizumab and long term prednisolone. In September 2021, after presenting with left lower lobar collapse, she underwent a flexible bronchoscopy which showed the presence of an inflammatory membrane occluding the bronchus to her left lower lobe. This was easily cleared and copious volumes of mucus were suctioned. No features of polychondritis or bronchomalacia were found. Biopsies of the membrane did not show any specific pattern of inflammation. Ongoing inflammatory changes were also found in the bronchus to the left upper lobe but without any obstruction to the airways. The airways in her right lung were noted to be completely normal. At first presentation maintenance prednisolone dose was increased from 10 mg OD to 20 mg OD and mycophenolate from 500 mg BD to 1 g BD. She continued to take monthly infusions of tocilizumab.

**Fig. 1:**
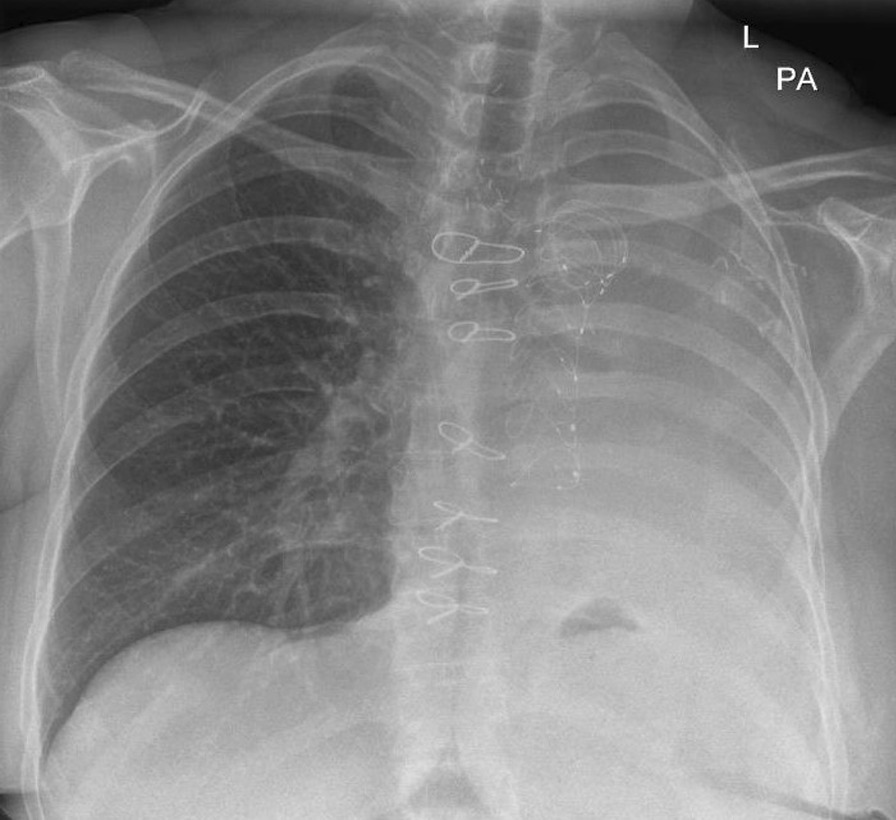
PA plain film chest radiograph illustrating complete white out of the left hemi-thorax suggestive of complete left sided collapse

**Fig. 2:**
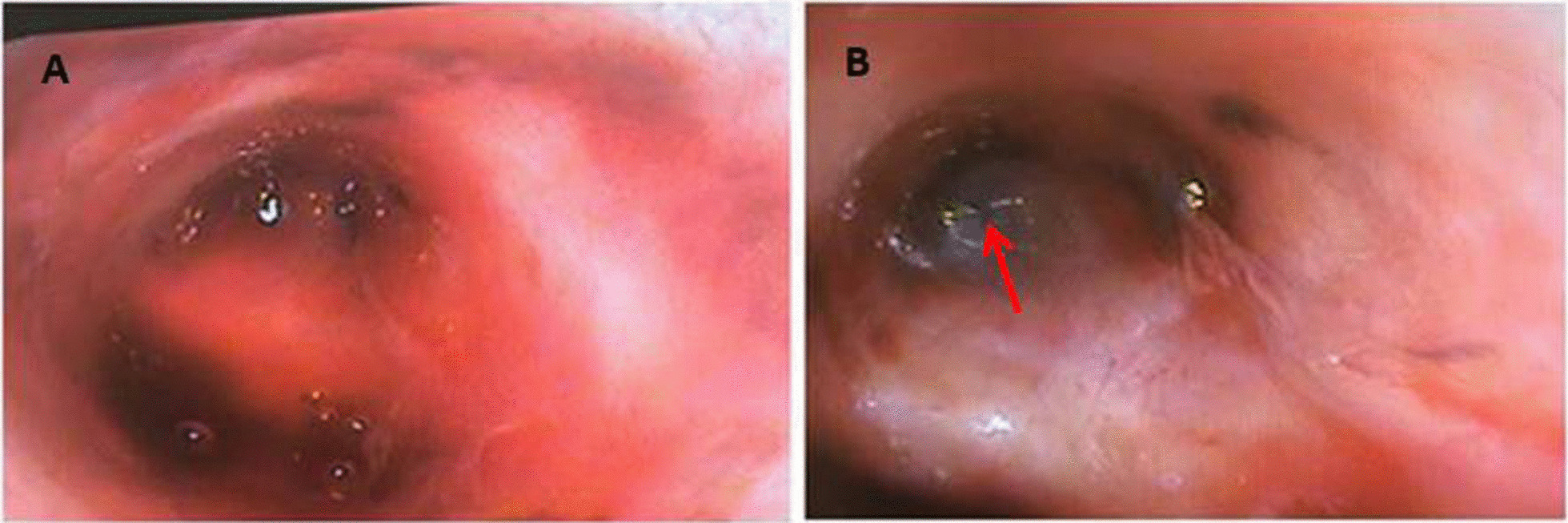
Bronchoscopic findings in February 2022 identified inflammatory membranes occluding both the left upper and lower lobe bronchi

The following year in February 2022 she again re-presented with worsening breathlessness. A chest x-ray showed a complete white out of her left lung (Fig. 1). A subsequent rigid and flexible bronchoscopy showed the presence of a thick, hyaline, inflammatory membrane occluding both the upper and lower lobe bronchi separately (Fig. 2A). The masses were also noted to be pulsatile and bled quite easily, and on further interrogation of the imaging, it was apparent the truncus arteriosus branches of the left pulmonary artery to the upper lobe were millimetres behind these membranes. The patient was started on a regimen of methylprednisolone and a bronchoscopy was performed a week later after symptomatic improvement in her breathlessness. Repeat bronchoscopy this time showed bulging of the membranous bronchus to the left upper and lower lobes. Significantly, the struts of FET could be seen invading through the bronchus to the upper lobe (red arrow, Fig. 2B).

**Fig. 3:**
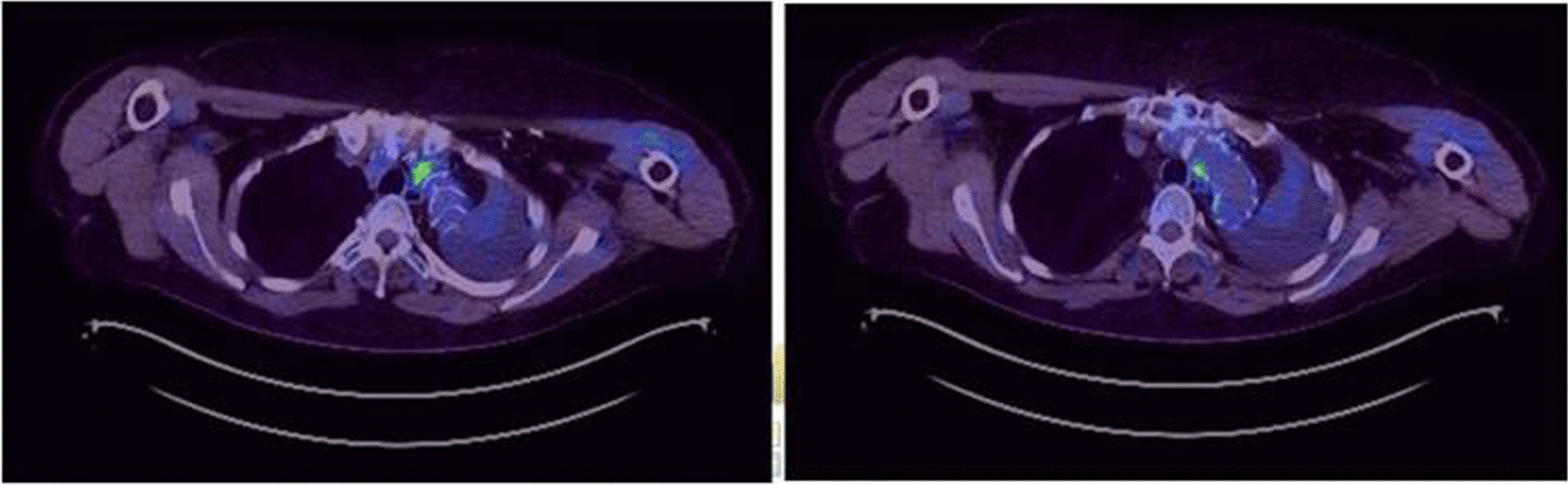
Axial slices of PET CT in March 2022 showed foci of increased uptake concerning for a graft infection. Ongoing complete left sided collapse of the lung is also present

**Fig. 4:**
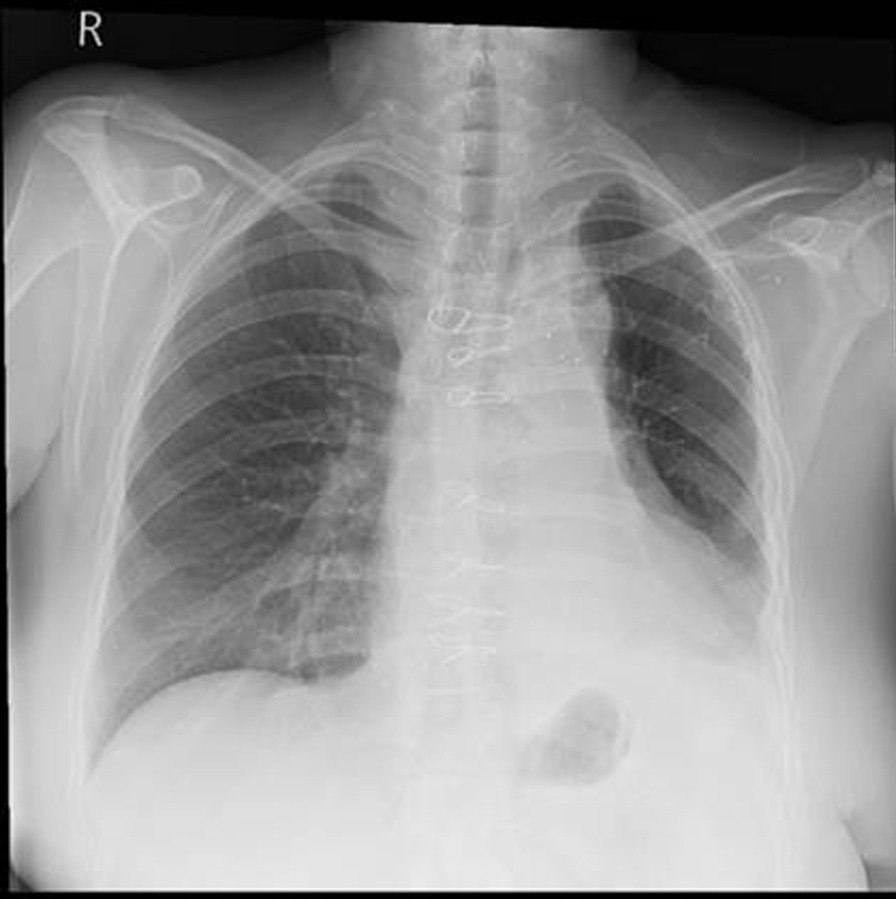
Chest x-ray prior to hospital discharge from July 2022, resolution of left upper lobe collapse

A PET CT Scan in March showed features concerning of an aortic graft infection and complete collapse of the left lung (Fig. 3). The patient returned to hospital in May for a replacement of her infected FET and repair of her aorto-bronchial fistula. During the operation it was noted that the intima of the aorta in the region of the left main bronchus was thickened and inflammatory. No attempt was made to dissect the bronchus given the potential of fistulation and surrounding tissue fragility. On completion of the operation, bronchoscopy showed a patent left upper lobe bronchus but an oedematous, occluded left lower lobe bronchus. On discharge she was noted to have an isolated left lower lobe collapse on her chest x-rays (Fig. 4).

**Fig. 5:**
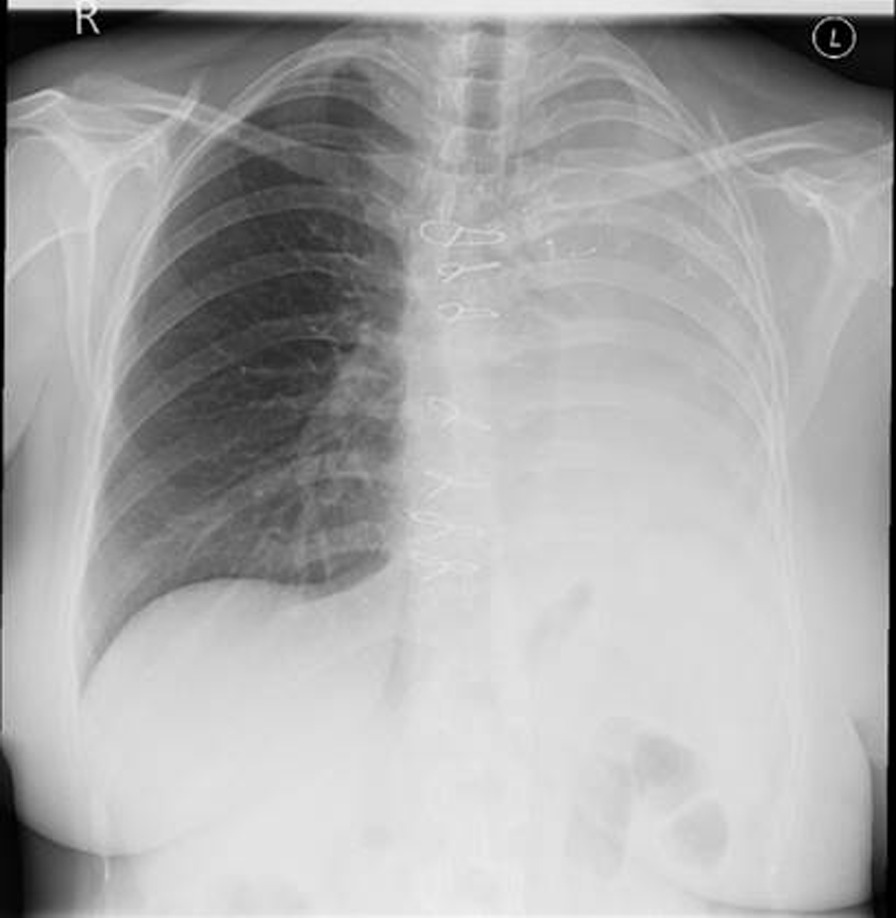
Chest x-ray one month post hospital discharge from August 2022 (re-collapse of upper lobe)

**Fig. 6:**
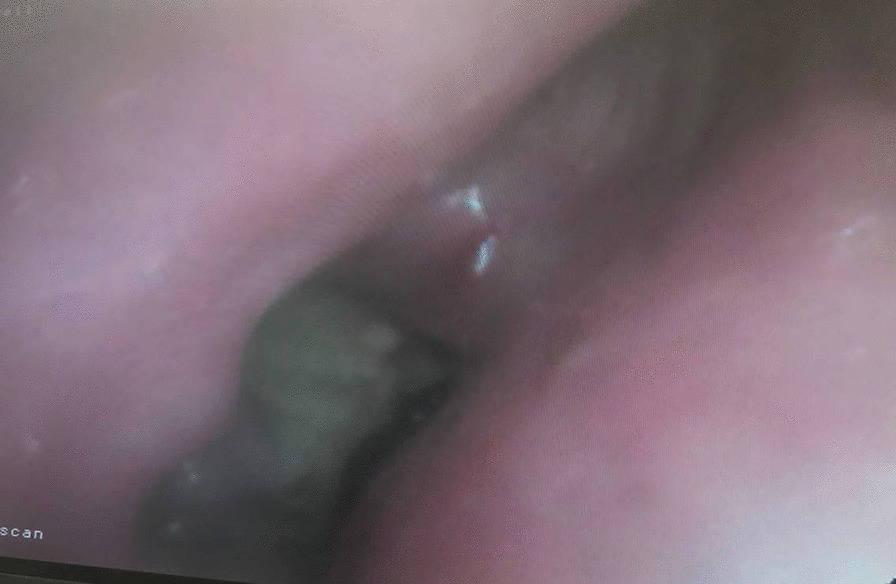
Bronchoscopy September 2022 showed complete re-occlusion of left main bronchus with a thick inflammatory exudate

Follow up in August with a chest x-ray and CT scan showed re-collapse of her left lung, with evidence of her left main bronchus being occluded (Fig. 5). Another bronchoscopy was arranged in September 2022 which showed that her left main bronchus was completely blocked with a hard inflammatory exudate (Fig. 6). It was not possible to dislodge the material with saline injections and it was also not possible to identify the LUL or LLL take-offs as the obstruction was proximal to the secondary carina. Given the recurrence of her condition, a pneumonectomy was considered. However, since the patient felt quite well in herself it was decided to continue managing her obstruction conservatively. Furthermore, there would be a high risk of stump failure with a left pneumectomy as the patient is on a significant immunosuppressive regime.

## Comment

We believe this case will add to the literature on the possible complications of Behcet’s disease / MAGIC Syndrome. In this case we highlighted two separate complications: formation of an aorto-bronchial fistula from herniation of the FET into the left main bronchus and the development of multiple endobronchial webs in the left lung causing recurrent lobar collapse.

In general, aneurysmal disease is challenging to treat surgically in Behcet’s disease due to a high rate of graft failure and recurrence [8, 9]. Though quite rare, aorto-bronchial fistulas are noted to be most frequently observed after surgical repair of aortic aneurysms [10]. Specifically in relation to a FET, the literature regarding its adverse complications is limited [11]. However, a recent large multicentre review of 437 patients who underwent a FET procedure showed that around 23% required a further aortic operation on follow up [12]. The most common reason in the aneurysmal cohort was due to enlargement of the distal thoracoabdominal aorta. Unfortunately, no data was presented regarding occurrence of stent migration, herniation, ABF formation or infection. We therefore believe this case will add to the current literature regarding complications of aneurysmal disease in Behcet’s.

There are also very few cases in the literature that describe the presence of endobronchial lesions in patients with Behcet’s. There have been reports of patients developing aphthous ulcers in the larynx leading to luminal stenosis [13]. Most relevant to our case, there has been one report of a patient with Behcet’s developing endobronchial granulomatosis in the right upper lobe bronchus and bronchus intermedius leading to severe bronchial stenosis [14]. Similar to our case, recurrence of stenosis was observed after initial recanalisation. However, this study reported some success in the use of Neodym-YAG laser resection with immunosuppression to achieve recanalisation. In our case, we believe it was prudent to manage these endobronchial lesions conservatively given their proximity to the pulmonary artery and the associated risk of haemorrhage. We believe this case highlights the multi-system nature of Behcet’s as well as demonstrating the rarity of some of the possible complications that could be associated with the disease. A multi-disciplinary team approach is of paramount importance in order to optimally manage patients with Behcet’s disease, balancing immunosuppressive regimens that need close monitoring and titration in the context of potential surgical intervention and the risk for intercurrent infection.

## Data Availability

All data and materials are available upon reasonable request from the corresponding author.
